# Nocturnal haemoglobin oxygen desaturation in urban and rural East African paediatric cohorts with and without sickle cell anaemia: a cross-sectional study

**DOI:** 10.1136/archdischild-2014-306468

**Published:** 2015-12-23

**Authors:** VS L'Esperance, T Ekong, SE Cox, J Makani, CR Newton, D Soka, A Komba, FJ Kirkham, CM Hill

**Affiliations:** 1 Division of Clinical Experimental Sciences, Faculty of Medicine, University of Southampton, Southampton, UK; 2 MRC International Nutrition Group, London School of Tropical Medicine and Hygiene, London, UK; 3 Muhimbili Wellcome Programme, Muhimbili University of Health and Allied Sciences, Dar-es-Salaam, Tanzania; 4 Neuroassessment department, KEMRI-Wellcome Trust Research Programme, Centre for Geographic Medicine Research (Coast), Kilifi, Kenya; 5 Department of Psychiatry, University of Oxford, Oxford, UK; 6 Neurosciences Unit, UCL Institute of Child Health, London, UK

**Keywords:** anaemia, sickle cell, haemoglobin oxygen saturation, Sleep

## Abstract

Low haemoglobin oxygen saturation (SpO_2_) predicts complications in children with sickle cell anaemia (SCA) in the North but there are few data from Africa, where the majority of the patients reside. We measured daytime and overnight SpO_2_ in children with SCA in routine follow-up clinic, and controls without symptoms of SCA, comparing rural (Kilifi, Kenya) and urban (Dar-es-Salaam, Tanzania) cohorts. Daytime SpO_2_ was lower in 65 Tanzanian children with SCA (TS; median 97 (IQR 94–100)%); p<0.0001) than in 113 Kenyan children with SCA (KS; 99 (98–100)%) and 20 Tanzanian controls (TC; 100 (98–100)%). Compared with 95 Kenyan children with SCA, in 54 Tanzanian children with SCA and 19 TC who returned for overnight oximetry, mean (KS 99.0 (96.7–99.8)%; TS 97.9 (95.4–99.3)%; TC 98.4 (97.5–99.1)%; p=0.01) and minimum nocturnal SpO_2_ (92 (86–95)%; 87 (78.5–91)%; 90 (83.5–93)% p=0.0001) were lower. The difference between children with SCA persisted after adjustment for haemoglobin (p=0.004). Urban Tanzanian children, with and without SCA, experience greater exposure to low daytime and night-time SpO_2_ compared with rural Kenyan children with SCA. Possible explanations include differences in the prevalence of obstructive sleep apnoea or asthma, alterations in the oxyhaemoglobin desaturation curve or cardiovascular compromise, for example, to shunting at atrial or pulmonary level secondary to increased pulmonary artery pressure. The fact that non-SCA siblings in the urban area are also affected suggests that environmental exposures, for example, air pollution, nutrition or physical exercise, may play a role. Further studies should determine aetiology and clinical relevance for the SCA phenotype in children resident in Africa.

**What is already known on this topic**

- Low daytime haemoglobin oxygen saturation is common in sickle cell anaemia, and may be exacerbated overnight by dips secondary to obstructive and central apnoea.
- Low haemoglobin oxygen saturation appears to be associated with complications of sickle cell anaemia including central nervous system events.
- Markers of severity of sickle cell anaemia, including abnormally high transcranial Doppler velocities and left ventricular hypertrophy, are associated with low haemoglobin oxygen saturation.

**What this study adds**

- In sickle cell anaemia, compared with rural Kenyan children, urban Tanzanian children had low daytime and mean and minimum overnight haemoglobin oxygen saturation.
- Sibling controls in Tanzania also had low daytime and mean and minimum overnight haemoglobin oxygen saturation, consistent with environmental factors in the aetiology.
- Haemoglobin levels were similar in the Tanzanian and Kenyan children with sickle cell anaemia and the difference in haemoglobin oxygen saturation survived adjustment for haemoglobin.

## Introduction

Homozygous sickle cell anaemia (SCA) is one of the most common monogenetic conditions in the world, with more than 70% of sufferers living in sub-Saharan Africa. Approximately 98% of oxygen carried in the blood is transported by haemoglobin with the remainder dissolved in plasma. The dysfunctional haemoglobin and right-shifted oxyhaemoglobin dissociation curve in SCA affect haemoglobin oxygen saturation, which can be measured by pulse oximetry (SpO_2_). Children spend half their lives asleep; sleep is associated with a natural fall in SpO_2_ as minute ventilation falls. Daytime and night-time (episodic and continuous) desaturation is common in Western populations with SCA and predicts complications.

In a Jamaican study, rural SCA populations achieved higher physical and mental health scores, with fewer limitations in daily living activities and better quality of life.^1^ In non-SCA adults enrolled in the Sleep Heart health study, sleep-disordered breathing and nocturnal hypoxaemia correlated with short-term changes in particulate matter pollutant level.^2^ Even though the major burden of SCA remains in sub-Saharan Africa, where air quality in cities is often poor (http://www.unep.org/transport/pcfv/PDF/DART-OVERVIEWAIRPOLLUTION.pdf; http://www.unep.org/urban_environment/PDFs/EABAQ2008-ProgressregionKeziaMbwambo.pdf),^3^ few studies have compared childhood SpO_2_ in urban with rural paediatric SCA populations in the African setting, particularly with respect to sleep.

Overnight pulse oximetry with signal extraction technology (Masimo) provides an inexpensive and accessible method of recording nocturnal SpO_2_. The objective of this study was to characterise daytime and overnight SpO_2_ in African children with SCA and compare those living in rural and urban settings.

## Methods

The study was approved by the Kenya National Ethical committee (SCC 688) and the Muhimbili University College of Health Sciences research committee (MU/RP/ AECNoI.XII/77). Children were recruited from confirmed HbSS, but otherwise unselected, cohorts of patients attending outpatient clinics in rural Kenya (Kilifi) in 2004^4^ and urban Tanzania (Dar-es-Salaam) in 2009.^5^ In Tanzania, household controls without symptoms of SCA were also recruited.^5^ Blood samples and pulse oximetry were obtained >12 weeks post-transfusion, and >12 weeks following acute illness. Resting daytime and overnight recordings of SpO_2_ were recorded by pulse oximetry (Masimo Irvine, California, USA) using a 2 s averaging time continuously during sleep. The data were analysed using Download 2001 software (Stowood Scientific, UK). Any residual movement artefact was excluded manually. We examined the overnight data for mean SpO_2_, and percentage of time spent with SpO_2_<90%.

### Data entry and analysis

Analyses were performed using the statistical software package SPSS V.21.0. χ^2^ was used to compare prevalence of symptoms of sleep-disordered breathing. Non-parametric statistics (median and IQR as descriptives; Kruskal–Wallis test for comparison between groups) are reported for non-normally distributed variables. A two-tailed p value of p<0.05 was considered to be significant. Scheffe's test was used for post hoc testing to compare means of the Kenyan and Tanzanian children with SCA and the controls.

## Results

**Figure 1:**
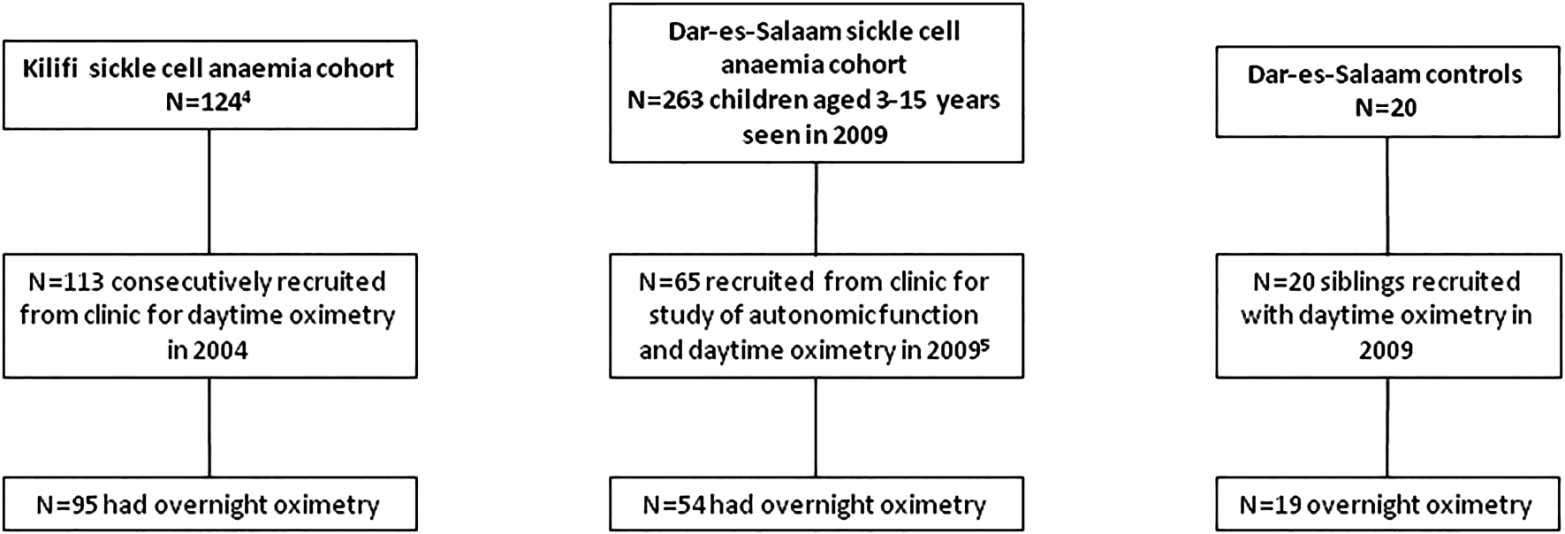
Flowchart for studies in Kilifi, Kenya in 2004 and Dar-es-Salaam, Tanzania in 2009.

The study included 113 Kenyan children with SCA (median age 6.4 years) consecutively recruited at Kilifi District Hospital in the first half of 2004 from a cohort of 124 (median age 6.3 years)^4^ and 65 (median age 7.3 years) recruited^5^ from a cohort of 263 potentially eligible Tanzanian children with SCA without stroke aged 3–16 years (median 10.1 years) seen in clinic from mid-March to mid-June 2009 (table 1; figure 1). The study was not offered to all potentially eligible Tanzanian children with SCA because of lack of equipment during the study period but there was no attempt to recruit children with specific symptoms, for example, respiratory or snoring. Additionally, 20 Tanzanian children without symptoms of SCA were assessed in 2009 (figure 1).^5^

**Table 1:** Differences between rural children with SCA and urban children with and without SCA

	Urban Tanzanian Non-SCA Controls (n=19 [20]) Median (IQR)	Urban Tanzanian SCA (n=54 [65]) Median (IQR)	Rural Kenyan SCA (n=95 [113]) Median (IQR)	P* for comparison between groups	P† for comparison with Tanzanian patients with SCA and controls	P† for comparison between Tanzanian and Kenyan patients with SCA
Age (years)	8.0 (4.0–10.4)	7.3 (5.1–10.6)	6.4 (3.7–10.1)	0.216		
	[8.7 (5.5–10.8)]	[7.1 (5.1–10.6)]	[6.5 (1.3–16.5)]			
BMI z-scores	−0.11 (−0.97 to 0.66)	−0.98 (−1.89 to 0.052)	−1.3 (−2.1 to −0.6)	0.005	0.4	0.059
	[−0.31 (−1.48 to 0.08)]	[−0.93 (−1.68 to 0.88)]	[−1.33 (−2.04 to 0.58]			
Haemoglobin (g/dL)	NA	7.1 (6.6–7.8) [7.1 (6.6–7.8]	7.5 (6.8–8.2) [7.4 (6.8–8.2)]	0.076		
Median daytime haemoglobin Oxygen saturation (%)	100 (98–100) [100 (98–100)]	97 (94.8–99.1) [97 (94–100)]	99 (98.0–100) [99 (98.0–100)]	0.001 [0.0005]	0.018 [0.001]	0.002 [0.0005]
Median overnight haemoglobin Oxygen saturation (%)	98.6 (97.5–99.1)	97.7 (95.1–99.3)	99.0 (96.7–99.8)	0.01	0.3	0.048
Median minimum haemoglobin Oxygen saturation (%)	90 (83.5–93)	87 (78.5–91)	92 (86–95)	0.001	0.2	0.003
Median overnight heart rate (bpm)	88.0 (81.9–97.5)	93.3 (85.2–102.9)	90.2 (79.1–102.1)	0.3		

Values in square brackets for whole cohort recruited.

*Kruskal–Wallis test.

†Post hoc comparison using Scheffe's test.

BMI, body mass index; SCA, sickle cell anaemia.

Mean daytime SpO_2_ was significantly lower in the urban Tanzanian children with SCA compared with the rural Kenyan children with SCA, including after adjustment for haemoglobin and nutritional status, which varied between groups (p=0.01) (table 1).

Ninety-five Kenyan and 54 Tanzanian children with SCA and 19 Tanzanian controls returned for overnight oximetry (figure 1). Table 1 shows age, body mass index-z-scores, haemoglobin, and daytime and overnight oximetry measures in the three groups. There was no difference in the prevalence of snoring at least half the week in the children with SCA who attended for overnight pulse oximetry compared with those who did not in either the Kenyan or the Tanzanian SCA cohort (χ^2^ p=0.6 and 0.7, respectively). Overnight mean SpO_2_ was significantly lower in urban Tanzanian children with SCA compared with rural Kenyan children with SCA (table 1), including after adjustment for haemoglobin (p=0.004), and was similar to Tanzanian controls. Similarly, minimum SpO_2_ was lower in urban Tanzanian children with SCA than in rural Kenyan children with SCA (table 1).

## Discussion

Urban children with SCA in Tanzania experience greater exposure to intermittent and continuous nocturnal haemoglobin oxygen desaturation in comparison with rural Kenyan children with SCA despite similar haemoglobin levels and both living at sea level. This may indicate that these two otherwise similar East African coastal populations may follow a different clinical course of disease, perhaps related to genetic or environmental factors such as nutrition or pollution.^3^

The aetiology of the differences in haemoglobin oxygen saturation is not clear. The α-thalassaemia 3.7 deletion is associated with daytime and nocturnal SpO_2_ in the Tanzanian SCA population, but this is unlikely to explain the difference as the prevalence is very similar in the two SCA populations (Williams-TN, personal communication). Obstructive sleep apnoea is a possible explanation. The lack of difference in symptom prevalence and absence of overweight children in the samples provide no positive evidence and central apnoeas are also relatively common in SCA; investigation of their relative importance with polysomnography in this setting would require considerable funding. Asthma and previous acute chest syndrome might play a role, but are rare in African settings and were not diagnosed in our patients. A vascular effect of air pollution,^6^ for example, increased pulmonary artery pressures with right-to-left shunting, might be an explanation in the context; the prevalence of high tricuspid jet velocities is similar to studies in the North in Dar-es-Salaam^7^ but there are currently no data for Kilifi. There might also be differences in the oxyhaemoglobin dissociation curve, although both Kilifi and Dar-es-Salaam are at sea level and in fact, exposure to carbon monoxide in polluted air may ameliorate SCA,^6^ at least in part by shifting the curve, typically right shifted in SCA, to the left. Another possible explanation is that living in a rural environment provides an opportunity for greater time spent in the natural environment engaging in unstructured outdoor activities, shown to be associated with physical health benefits in children, while children in a rural environment may also be less exposed to pollutants than those living in rapidly developing cities such as Dar-es-Salaam.^3^

A limitation of this study is the potential for a recruitment bias, as families may prioritise an opportunity to participate in research if they are concerned about their children's health, and to return for overnight oximetry if concerned about their sleep. Although the majority of Kenyan clinic population was studied, for the larger Tanzanian population, a convenience sample of consecutive patients with SCA and household controls was recruited from clinic over a 3-month period. Tanzanian children participating were a little younger than the remainder seen in clinic, comparable with the Kenyan patients with SCA and mainly in the age group most vulnerable to sleep-disordered breathing. Confidence that this is a real difference between populations is increased by the differences in daytime SpO_2_ in the larger clinical samples. The majority of the Tanzanian children recruited returned for overnight pulse oximetry; those returning did not have more clinical evidence for sleep-disordered breathing than those who did not.

In summary, urban Tanzanian children, with and without SCA, experience greater exposure to low daytime and night-time SpO_2_ compared with rural Kenyan children with SCA. The aetiology of the desaturation is uncertain but differences in air quality might be a factor. The clinical relevance in an African setting requires further investigation.
